# High-throughput quantification of aflatoxin in moldy peanuts using hyperspectral imaging and CNN: Comparative evaluation of machine learning algorithms and deep learning models

**DOI:** 10.1016/j.crfs.2025.101209

**Published:** 2025-09-24

**Authors:** Min Pang, Yingge Wang, Mengke Li, Li Xu, Chun Gao, Shaotong Jiang, Zhi Zheng, Lili Cao

**Affiliations:** a School of Food and Bioengineering, Hefei University of Technology, Hefei, 230009, PR China; b Key Laboratory for Agricultural Products Processing of Anhui Province, Hefei, 230009, PR China; c Anhui Jiexun Optoelectronic Technology Co., Ltd, Hefei, 230000, PR China

**Keywords:** Aflatoxin, Hyperspectral imaging, Deep learning, Correlation analysis, Non-destructive technologies

## Abstract

Aflatoxin contamination in peanuts poses serious health risks, requiring rapid, non-destructive detection methods. This study developed a hyperspectral imaging (HSI) approach combined with deep learning for quantitative aflatoxin analysis in moldy peanuts. The dynamic degradation of nutrients during mold growth was monitored, and correlations between physicochemical properties, aflatoxin levels, and spectral features were investigated. Various preprocessing methods and feature selection techniques were compared, evaluating conventional machine learning (Partial Least Squares Regression (PLSR), Random Forest (RF), Least Absolute Shrinkage and Selection Operator (LASSO)) against a convolutional neural network (CNN). The CNN model, optimized with median filtering and genetic algorithm-based feature selection, achieved superior performance (R^2^p = 0.972, RMSEp = 8.203, RPDp = 2.738). The proposed HSI-CNN framework provides an efficient, non-destructive solution for high-throughput aflatoxin quantification, supporting industrial food safety monitoring.

## Highlights

• HSI was coupled to DL for aflatoxin detection.
• CNN outperforms traditional ML methods.
• MF and GA optimizes spectral feature selection.
• HSI-CNN demonstrates industrial application potential.

## Graphical abstract

**Figure:**
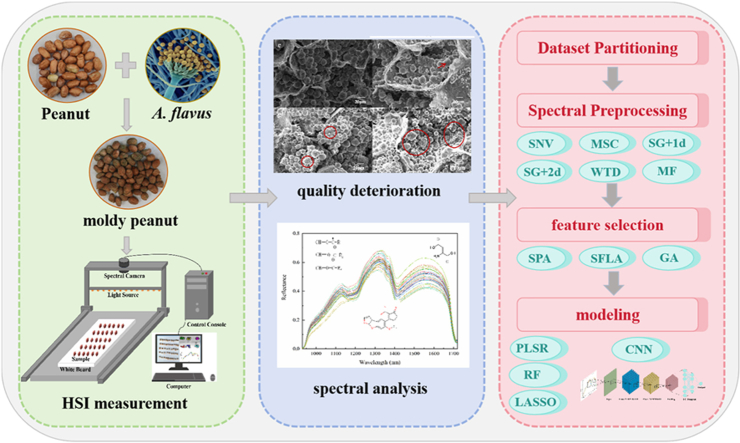

## 1 Introduction

Peanuts are a globally significant economic crop and a valuable nutritional food source, rich in protein, unsaturated fatty acids, vitamin E, and various essential minerals. They play a crucial role in both human diets and agricultural economies (J. Sun et al., 2020). The nutritional components of peanuts offer potential health benefits, including regulation of blood lipids, antioxidant effects, and a reduced risk of cardiovascular diseases. However, peanuts are particularly vulnerable to contamination by *Aspergillus flavus* during cultivation, storage, and processing. This fungus produces highly carcinogenic secondary metabolites known as aflatoxins (AFTs). Aflatoxin B_1_ (AFB_1_), classified as a Group 1 carcinogen by the International Agency for Research on Cancer (IARC), can accumulate in the human body through the food chain, posing serious health risks, including hepatotoxicity, immunosuppression, and hepatocellular carcinoma (Liu et al., 2022). Stringent regulatory limits have been set worldwide: In China, AFB_1_ levels in food and feed must not exceed 20 μg/kg, while the European Union imposes a stricter limit of 8 μg/kg for peanuts intended for food use. The U.S. Department of Agriculture enforces a maximum permissible level of 10 μg/kg in food products (Tao et al., 2022).

Aflatoxin detection currently faces several challenges. While conventional methods such as high-performance liquid chromatography (HPLC) (Turksoy and Kabak, 2020)and enzyme-linked immunosorbent assay (ELISA) (He et al., 2024) offer reliable accuracy, they require complex sample preparation procedures and are inherently destructive, making them unsuitable for high-throughput or real-time monitoring applications (Guo et al., 2020). Furthermore, the heterogeneous distribution of aflatoxins in peanut matrices increases the risk of sampling errors, and the trace-level contamination (at μg/kg concentrations) necessitates exceptionally stringent sensitivity requirements for detection methods.

In recent years, hyperspectral imaging (HSI) technology has shown significant potential in agricultural product quality and safety monitoring due to its unique advantages. By integrating spectral analysis with image processing, HSI captures both spatial information and continuous narrow-band spectral characteristics, forming a three-dimensional “image-spectrum fusion” data cube (x, y, λ). This technique not only preserves the morphological features of samples but also detects subtle biochemical compositional changes, thus providing multidimensional information for non-destructive detection (Sun et al., 2019). Specifically, for aflatoxin detection, HSI can identify characteristic spectral responses related to biochemical alterations in peanut tissues caused by fungal contamination, including diagnostic absorption peaks associated with protein denaturation and lipid oxidation (Kim et al., 2023). With advancements in artificial intelligence, deep learning approaches have proven to be highly effective in spectral data analysis (Yuan et al., 2022). Compared to traditional machine learning algorithms, deep learning models (e.g., convolutional neural network, CNN) can autonomously extract high-level features from hyperspectral data through multiple nonlinear transformations, thus eliminating the need for manual feature engineering (Zhang et al., 2025). The unique properties of CNN, including local connectivity, weight sharing, and pooling operations, enable them to have a strong representational capacity for joint spatial-spectral features in hyperspectral data. The architectural advantages of CNN are inherently well-suited for the spatial-spectral characteristics of HSI cubes. Specifically, the property of local connectivity allows the network to efficiently extract localized spatial features from adjacent pixels within each spectral band. Simultaneously, weight sharing significantly reduces model complexity and mitigates the risk of overfitting, which is crucial given the high-dimensional nature of HSI data where the number of spectral variables often far exceeds the number of samples.

Existing studies on food quality detection have investigated various commodities, including fruits and vegetables (Tunny et al., 2023; B. Li et al., 2024), cereals (Vega-Castellote et al., 2025), and meat products (Wei et al., 2024). However, most hyperspectral imaging (HSI) research in this field relies on shallow machine learning (ML) models, such as partial least squares (PLS) (F. Li et al., 2024), support vector machines (SVM) (Jin et al., 2023), and artificial neural networks (ANN) (Choi et al., 2024). Notably, there is a lack of in-depth analysis regarding (1) the dynamic correlation between nutritional deterioration and spectral characteristics during peanut mold development, and (2) robust quantitative modeling of aflatoxin contamination levels in peanuts. To address these gaps, the present study integrates HSI with deep learning algorithms to establish a rapid, non-destructive quantitative model for the detection of aflatoxin content in peanuts. By monitoring physicochemical and microstructural changes during mold growth, we analyze the corresponding variations in spectral absorption peaks to elucidate the relationships among biochemical properties, toxin concentrations, and diagnostic spectral bands. Furthermore, we systematically compare different spectral preprocessing methods and feature selection strategies to optimize the performance of the model. The ultimate goal is to develop an accurate, efficient, and non-destructive detection approach, which will provide new technical support for quality control in the peanut industry. In addition to its immediate application in food safety assurance, this research offers methodological insights that can be applied to contaminant detection in other agricultural products.

## 2 Materials and methods

### 2.1 Materials and equipment

The peanut variety used in the experiment was Northeast China white-shelled peanuts, purchased from the local supermarket. *A. flavus* 2219 strains were obtained from China Industrial Microbial Strain Preservation and Management Centre. The AFB_1_ standard substance was purchased from Yifang Technology Co, Ltd., with the purity over 99 % (HPLC). Acetonitrile and methanol were obtained from Macklin with purity over 98 % (Macklin Biotech Co, Ltd., China). All chemicals and organic solvents were of analytical grade and used as supplied. UltiMate 3000 High Performance Liquid Chromatography was purchased from Thermo Fisher Scientific Co, Ltd., SE206 Fat Analyzer was purchased from ALRVA Co, Ltd., K9840 Kjeldahl Nitrogen Analyzer was purchased from Hanon Group Co, Ltd.

### 2.2 Sample preparation

The purchased peanut samples were surface-sterilized by rinsing three times with 75 % (v/v) ethanol followed by air-drying at room temperature. Precisely 45 g aliquots of peanuts were weighed using an analytical balance (accuracy ±0.1 mg), totaling 36 experimental samples. The samples were incubated at a constant temperature of 28 °C (±0.5 °C) under three different water activity (aw) conditions (0.90, 0.93, and 0.96 aw). During the 21-day incubation period, random sampling was conducted at two-day intervals to monitor the progression of fungal growth and aflatoxin production, with triplicate samples collected at each time point for analytical measurements. The experimental design ensured systematic tracking of biochemical changes while maintaining controlled environmental parameters throughout the incubation process.

### 2.3 HPLC analysis of aflatoxins

The aflatoxin content in peanut samples was determined according to GB/T 36858-2018 using high-performance liquid chromatography (HPLC). A series of AFB_1_ standard solutions were prepared in acetonitrile at concentrations of 1, 5, 10, 20, 50, and 100 ng/mL to establish the calibration curve. The peanut samples were finely ground and subjected to extraction and derivatization procedures to isolate aflatoxins prior to analysis. Chromatographic separation was achieved using a Venusil MP C18 (2) analytical column (4.6 mm × 250 mm, 5 μm particle size) maintained at 30 °C. The mobile phase consisted of methanol/acetonitrile/water (20v/10v/70v) delivered at a flow rate of 1.0 mL/min. Fluorescence detection was performed with excitation and emission wavelengths set at 365 nm and 440 nm, respectively, for optimal AFB_1_ quantification.

### 2.4 Physicochemical property analysis

The physicochemical characteristics of peanut samples were determined using standardized analytical methods as follows: Water content was measured according to GB 5009.3-2016 (“Determination of water in foods”) employing the direct drying method. Fat content analysis was performed using the Soxhlet extraction method specified in GB 5009.6-2016 (“Determination of fat in foods”). Protein content was quantified using the Kjeldahl nitrogen determination method as prescribed in GB 5009.5-2016 (“Determination of protein in foods”). Acid value was determined through petroleum ether extraction following GB/T 5510-2011 (“Grain and oil inspection - Determination of fatty acid value in cereals and oilseeds").

### 2.5 Scanning Electron Microscopy (SEM) sample preparation

The peanut samples were carefully sectioned into slices measuring approximately 3 mm (length) × 2 mm (width) × 1 mm (thickness) using a precision blade. The prepared samples were then transferred to centrifuge tubes and completely immersed in n-hexane for 8 h at room temperature (25 ± 2 °C) to facilitate lipid extraction. Following this treatment, the lipids were removed and excess solvent was absorbed using filter paper. To ensure complete removal of residual n-hexane, a solvent exchange protocol was implemented. Samples were sequentially immersed in anhydrous ethanol for 15-min intervals, with this process repeated three times. After the final ethanol wash, samples were air-dried in a fume hood for 2 h to allow complete solvent evaporation. Prior to microscopic examination, the processed samples underwent lyophilization for 12 h (−50 °C, 0.05 mBar) to preserve structural integrity. The dehydrated samples were then mounted on aluminum stubs using conductive carbon tape and sputter-coated with gold to enhance conductivity. Microstructural characterization was performed using a field emission scanning electron microscope (COXEM, EM-30N) operated at 10 kV accelerating voltage with secondary electron detection.

### 2.6 Hyperspectral measurement

**Fig. 1:**
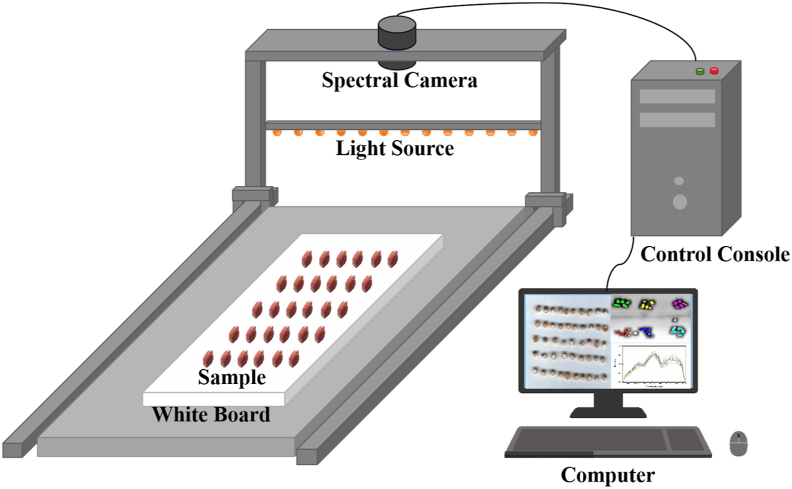
Schematic diagram of the hyperspectral imaging acquisition system.

The hyperspectral imaging system employed in this study consisted of an Image-λ “Spectral Imaging” series hyperspectral machine (Zolix Instruments Co., Ltd., China) operated using SpecVIEW software. As illustrated in Fig. 1, the system configuration comprised four principal components: a computer workstation for data acquisition and processing, a motorized translation stage for sample positioning, a line-scan camera system, and a uniform light source comprising a halogen light strip. Hyperspectral data collection was performed using a high-speed line-scanning camera (SPECIM FX17, Finland) operating in push-broom mode, with spectral coverage spanning 900–1700 nm (224 spectral bands) at a resolution of 8 nm. The camera was positioned at a fixed working distance of 28 cm from the sample plane, with a 35 mm focal length lens providing appropriate field of view. For radiometric calibration, a certified white reference panel (99 % reflectance) was used to establish baseline reflectance values. Prior to sample acquisition, dark current reference images (0 % reflectance) were obtained with the lens capped, while white reference images were collected using the calibration panel under identical illumination conditions (Liu et al., 2025). The raw hyperspectral data were normalized using Equation (1). Following calibration, region-of-interest (ROI) analysis was conducted using ENVI 5.6 software (Harris Geospatial Solutions). To ensure the representativeness and stability of the spectral data, a standardized protocol was applied for ROI selection. For each peanut kernel, three circular ROIs (diameter: ∼20 pixels) were systematically distributed across its surface, deliberately avoiding areas with obvious physical damage, shadows, or highlights. The mean spectrum from each ROI was calculated and recorded, providing three spectral replicates per sample for subsequent analysis. Representative spectral signatures were obtained by calculating the mean reflectance values across defined ROIs, generating characteristic spectral curves for subsequent chemometric analysis.

(1) $$\begin{array}{c} R_{1}=\frac{R_{0}-R_{B}}{R_{W}-R_{B}} \end{array}$$

where R_w_ is the white reference image obtained using a standard white Teflon tile (∼100 % reflectance) and R_B_ is the black reference image acquired by covering the camera lens completely with its own black cap (∼0 % reflectance).

### 2.7 Hyperspectral data preprocessing

Six distinct preprocessing methods were systematically applied to the acquired hyperspectral data to enhance spectral features and mitigate various types of noise:

(1) Standard Normal Variate (SNV)

SNV is a row-wise normalization method designed to mitigate spectral variations caused by physical effects, including particle size distribution, surface scattering, and path length differences. This preprocessing technique operates on individual spectra by centering and scaling each observation to achieve zero mean and unit variance. The formula for SNV is as follows:

(2) $$\begin{array}{c} x_{i}^{SNV}=\frac{x_{i}-\mu_{i}}{\sigma_{i}} \end{array}$$

where x_i_ represents the raw spectral measurement of the i-th band, μ is the mean reflectance value of the spectrum, and σ denotes its standard deviation.

(2) Multiplicative Scatter Correction (MSC)

MSC is a spectral preprocessing technique specifically designed to compensate for light-scattering artifacts induced by physical sample characteristics (D. Li et al., 2024). The method operates by aligning each individual spectrum to a reference mean spectrum through linear regression analysis, thereby isolating chemically relevant absorption features from physical interference effects. The formula for MSC is as follows:

(3) $$\begin{array}{c} x_{i}^{MSC}=\frac{b_{i}\cdot x_{ref}+e_{i}}{b_{i}} \end{array}$$

where x_i_ represents the raw spectral measurement of the i-th band,

(3) Savitzky-Golay Smoothing with First Derivative (SG-1d)

SG-1d is a widely adopted spectral preprocessing technique that simultaneously addresses noise reduction and spectral feature enhancement. For the SG-1d, a window size of 11 points and a polynomial order of 2 were applied. The formula for SG+1d is as follows:

(4) $$\begin{array}{c} x^{\prime }\left(\lambda_{i}\right)=\frac{\sum_{k=-m}^{m}c_{k}\cdot x\left(\lambda_{i+k}\right)}{\Delta \lambda } \end{array}$$

where c_k_ is SG convolution coefficients; 2m + 1 is window size; Δλ is wavelength interval.

(4) Savitzky-Golay Smoothing with Second Derivative (SG-2d)

SG-2d represents an advanced spectral preprocessing approach that simultaneously achieves noise suppression and enhanced feature resolution while maintaining critical peak morphology. For the SG-2d, a window size of 11 points and a polynomial order of 2 were applied. The formula for SG+2d is as follows:

(5) $$\begin{array}{c} x^{\prime \prime }\left(\lambda_{i}\right)=\frac{2\sum_{k=-m}^{m}c_{k}^{\left(2\right)}\cdot x\left(\lambda_{i+k}\right)}{{\left(\Delta \lambda \right)}^{2}} \end{array}$$

where $c_{k}^{\left(2\right)}$ are optimized coefficients for second derivative calculation.

(5) Wavelet Transform Denoising (WTD)

WTD is a multiscale signal processing technique that has demonstrated exceptional performance in spectral noise reduction while preserving critical chemical information. This method operates through a sophisticated decomposition-reconstruction framework that addresses the non-stationary nature of spectroscopic noise. The formula for WTD is as follows:

(6) $$\begin{array}{c} w_{j,k}=sign\left(w_{j,k}\right){\left(\left|w_{j,k}\right|-\lambda \right)}_{+} \end{array}$$

where w_j,k_ represents wavelet coefficients at scale j and position k, and λ is the threshold value.

(6) Median Filtering (MF)

MF is a nonlinear digital filtering technique that effectively suppresses impulse noise while preserving spectral edge features through order-statistic processing within a sliding window. Unlike linear smoothing methods, it demonstrates particular efficacy in handling salt-and-pepper noise common in hyperspectral imaging systems. The formula for MF is as follows:

(7) $$\begin{array}{c} {\hat{x}}_{i}=median\{x_{i-k},\cdots ,x_{i},\cdots ,x_{i+k}\} \end{array}$$

where the window width W = 2k+1 (typically odd-numbered).

### 2.8 Feature extraction and selection

Three distinct methods—Successive Projection Algorithm (SPA), Shuffled Frog Leaping Algorithm (SFLA), and Genetic Algorithm (GA)—were employed to select the most informative spectral bands. Each method independently identified 30 optimal wavelengths for subsequent modeling.

(1) Successive Projection Algorithm (SPA) is a mathematically rigorous variable selection method widely applied in spectroscopy, chemometrics, and multivariate calibration. Its core principle involves iteratively projecting spectral data to identify a subset of wavelengths with maximum information content and minimal redundancy (Y. Sun et al., 2020).
(2) Shuffled Frog Leaping Algorithm (SFLA) is a metaheuristic optimization algorithm inspired by the foraging behavior of frogs in wetlands. It combines: Local search (mimicking memetic algorithms) and Global information exchange (similar to Particle Swarm Optimization, PSO).
(3) Genetic Algorithm (GA) is a biologically inspired optimization technique that mimics the principles of natural selection to identify optimal spectral features. The algorithm encodes potential wavelength combinations as “chromosomes” and iteratively refines them through three fundamental evolutionary operators: Selection, where wavelengths with high fitness scores are preferentially retained; Crossover, which combines high-performing spectral bands from parent chromosomes to explore new feature combinations; Mutation, introducing random modifications to maintain population diversity and prevent premature convergence to local optima (Wang et al., 2024).

### 2.9 Modeling and model evaluation

#### 2.9.1 Conventional regression models

(1) Partial Least Squares Regression (PLSR)

PLSR is a multivariate statistical method designed to handle high-dimensional datasets where predictors exhibit multicollinearity or when the number of variables exceeds sample size (Shi et al., 2023). By integrating principles from principal component analysis (PCA) and multiple linear regression, PLSR identifies latent variables (LVs) that maximize covariance between spectral predictors (X) and aflatoxin content (Y). The parameters of PLSR used in this study are shown in Table S1.

(2) Random Forest (RF)

RF is an ensemble learning algorithm that constructs multiple decision trees through bootstrap sampling (bagging) and feature randomization. Each tree is trained on a random subset of data and wavelengths, with final predictions averaged across all trees to reduce variance (Malavi et al., 2024). RF excels at capturing nonlinear relationships and provides intrinsic feature importance rankings based on mean squared error (MSE) reduction. Despite its robustness against overfitting, the model's “black-box” nature and computational demands are notable limitations. The parameters of RF used in this study are shown in Table S2.

(3) Least Absolute Shrinkage and Selection Operator (LASSO)

LASSO is an improved method of linear regression proposed by Robert Tibshirani in 1996. It achieves feature selection and prevents overfitting by introducing L1 regularization, making it particularly suitable for high-dimensional data. Based on ordinary least squares (OLS), LASSO adds an L1 regularization term that compresses some regression coefficients to zero, thereby automatically selecting important variables, removing redundant features, reducing model complexity, and improving generalization ability (Ping et al., 2025). The parameters of LASSO used in this study are shown in Table S3.

#### 2.9.2 Deep learning model

**Fig. 2:**
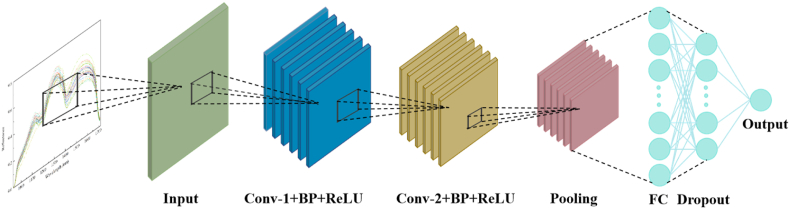
Schematic diagram of the convolutional neural network (CNN) architecture.

Convolutional Neural Network (CNN) is a specialized class of deep learning models particularly effective for processing structured grid-like data, such as spectral sequences, due to their ability to automatically extract hierarchical features through local receptive fields and weight-sharing mechanisms. In this study, we developed an optimized one-dimensional CNN architecture specifically designed for hyperspectral detection of aflatoxin contamination in peanuts (as illustrated in Fig. 2). The parameters of CNN are shown in Table S4. The model features a 7-layer network incorporating two 1D convolutional layers with 3 × 1 kernels and 16/32 filters respectively, followed by batch normalization and ReLU activation functions to enhance feature extraction stability and nonlinear representation. Notably, traditional fully connected layers were replaced with a global average pooling layer to significantly reduce model parameters while maintaining performance. To improve generalization capability, Gaussian noise (σ = 0.02) was added for data augmentation and a dropout rate of 0.3 was implemented to prevent overfitting. The model was trained using the Adam optimizer with an initial learning rate of 0.001 to ensure efficient convergence. The innovation lies in a tailored “shallow-but-wide” design that preserves subtle spectral features, combined with a novel triple regularization strategy (Global Average Pooling, high-intensity Dropout, and Gaussian noise augmentation) to effectively prevent overfitting on small-sample HSI data. This carefully designed architecture effectively addresses the unique characteristics of spectral data, achieving an optimal balance between feature abstraction capability and computational efficiency, while demonstrating strong robustness even with limited training samples.

#### 2.9.3 Model evaluation metrics

To comprehensively assess model performance, three key metrics were employed:

(1) Coefficient of Determination (R^2^)

R^2^ quantifies the proportion of variance in aflatoxin concentration explained by the model.

(8) $$\begin{array}{c} R^{2}=1-\frac{\sum_{i=1}^{n}{\left(y_{i}-{\hat{y}}_{i}\right)}^{2}}{\sum_{i=1}^{n}{\left(y_{i}-\bar{y}\right)}^{2}} \end{array}$$

where $y_{i}$ is the ith true value; ${\hat{y}}_{i}$ is the ith predicted value; $\bar{y}$ is the mean of the true values: $\bar{y}=\frac{1}{n}\sum_{i=1}^{n}y_{i}$; n is the number of samples.

(2) Root Mean Square Error (RMSE)

RMSE measures the average prediction error in original units (μg/kg).

(9) $$\begin{array}{c} RMSE=\sqrt{\frac{1}{n}\sum_{i=1}^{n}{\left(y_{i}-{\hat{y}}_{i}\right)}^{2}} \end{array}$$

where $y_{i}$ is the ith true value; ${\hat{y}}_{i}$ is the ith predicted value; n is the number of samples.

(3) Residual Prediction Deviation (RPD)

RPD evaluates model robustness by comparing prediction error to natural data variability.

(10) $$\begin{array}{c} RPD=\frac{SD}{RMSE} \end{array}$$

where SD is Standard Deviation of reference values, RMSE is Root Mean Square Error.

### 2.10 Correlation analysis

Pearson correlation analysis was performed to examine the relationships between the physicochemical properties, aflatoxin content, and characteristic wavelengths, with the results visualized as a heatmap. The Pearson correlation coefficient (r) measures the linear relationship between two continuous variables, ranging from −1 to 1. It is based on covariance (Cov) and standard deviations (σ), quantifying how variables vary together relative to their individual variability. The formula for r is as follows:

(11) $$\begin{array}{c} r_{X,Y}=\frac{Cov\left(X,Y\right)}{\sigma X\sigma Y}=\frac{\sum_{i=1}^{n}\left(X_{i}-\bar{X}\right)\left(Y_{i}-\bar{Y}\right)}{\sqrt{\sum_{i=1}^{n}{\left(X_{i}-\bar{X}\right)}^{2}}\sqrt{\sum_{i=1}^{n}{\left(Y_{i}-\bar{Y}\right)}^{2}}} \end{array}$$

where $\bar{X}$, $\bar{Y}$: sample means of X and Y; Cov: Covariance; σX, σY:Standard deviations of X and Y.

### 2.11 Software

The statistical analysis of the data was performed using IBM SPSS Statistics 27. All of the algorithms that are used in this study are executed in MATLAB R2023b.

## 3 Results and discussing

### 3.1 Dataset partitioning and aflatoxin content

**Fig. 3:**
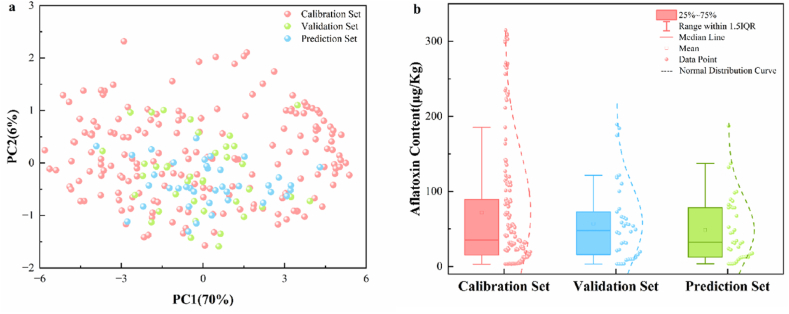
(a) PCA score plot showing data distribution partitioned by Kennard-Stone algorithm; (b) Box plots of aflatoxin concentrations in training, validation and prediction sets.

The dataset was partitioned using the Kennard-Stone algorithm, with visualization achieved through PCA dimensionality reduction, as shown in Fig. 3a. This method ensures that the selected samples uniformly span the entire multivariate space of the dataset, thereby providing a more representative calibration set and improving the robustness and predictive performance of the developed model compared to random splitting. The samples were divided into calibration (70 %), validation (15 %), and prediction (15 %) sets, ensuring that the training set comprehensively covered the entire data range, while the validation and prediction sets were appropriately contained within the training set's scope. The uniform spatial distribution, without isolated clusters, further confirmed the rationality of the dataset partitioning. HPLC analysis revealed that aflatoxin content ranged from 3.01 to 314.78 μg/kg for the calibration set, 3.25–189.34 μg/kg for the validation set, and 3.65–187.24 μg/kg for the prediction set (Fig. 3b), further validating that the training set fully encompassed the toxin concentration ranges of both the validation and prediction sets. This scientific partitioning approach ensured that the training process captured the complete data characteristics, while maintaining independence and reliability in the validation and prediction sets. Thus, it laid a solid foundation for accurate predictive modeling.

### 3.2 Physicochemical properties and microstructural changes of samples

**Fig. 4:**
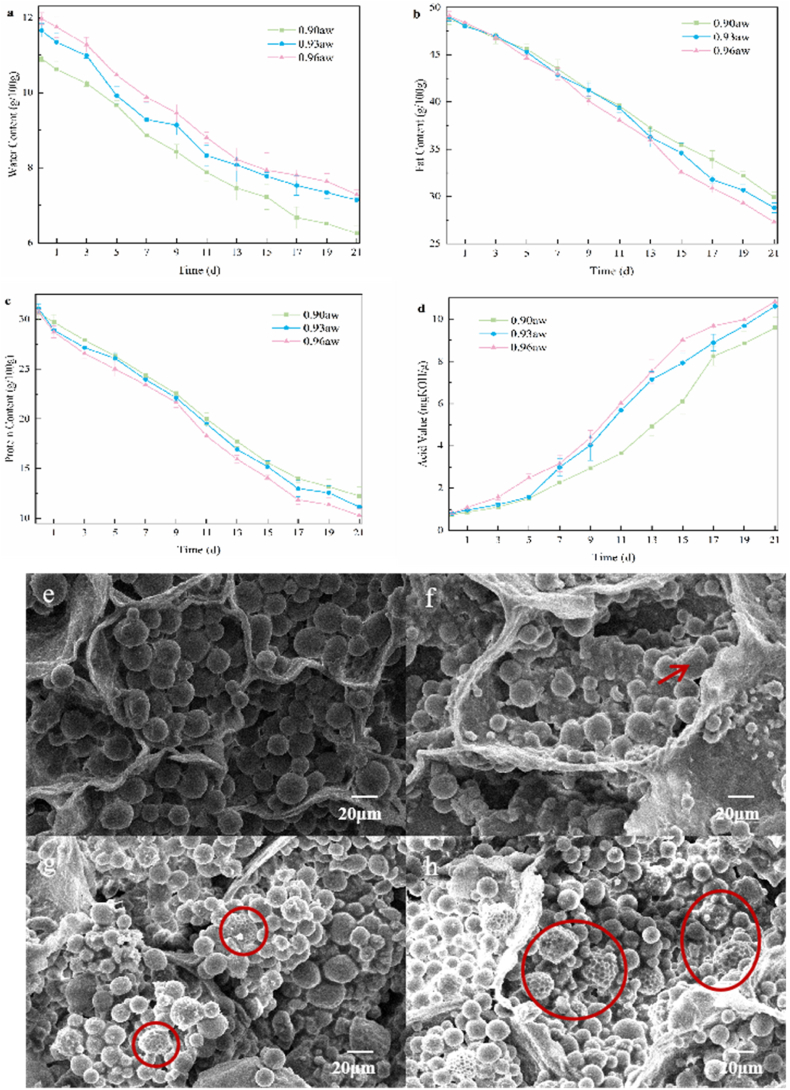
Changes in physicochemical properties and microstructure of peanut samples during storage: (a) water content, (b) fat content, (c) protein content, (d) acid value, (e) SEM image of control peanut section, (f) SEM image of peanut section after 5-day incubation, (g) SEM image of peanut section after 11-day incubation, (h) SEM image of peanut section after 21-day incubation.

As shown in Fig. 4a, significant differences in water content were observed among the three experimental groups. By day 7, water content of Group 2 samples had decreased to 9.28 g/100g, while Group 1 samples exhibited a gradual decline from 10.91 g/100g to 6.25 g/100g over the course of the incubation period. Fig. 4b illustrates a general decreasing trend in fat content during storage, with the group exhibiting the highest water activity showing the most pronounced reduction. Although the initial fat contents were comparable across the groups, distinct differences began to emerge by day 5. Group 3 samples experienced the most rapid fat degradation, reaching 44.66 g/100g, likely due to their higher water activity, which facilitated early mold colonization. Between days 7 and 15, lipid degradation accelerated, coinciding with the logarithmic growth phase of *A. flavus*, which metabolizes peanut lipids as a nutrient source (Guo et al., 2023). Later stages saw a moderation in lipid depletion rates. As shown in Fig. 4c, the protein content of the peanut samples exhibited an overall decreasing trend, with the third group samples showing a more rapid decline between days 9 and 17, decreasing from 23.41 g/100g to 11.87 g/100g. As seen in Fig. 4d, the acid value increased during incubation. In the early stages of incubation, the acid value changed slowly, but in the third group, the acid value began to increase significantly on day 3, reaching 1.57 mgKOH/g. In the second group, the acid value changed significantly after day 5, reaching 1.58 mgKOH/g, while the first group showed a slower change. High water activity (Group 3) induced a synergistic degradation pattern, where water redistribution created favorable conditions for mold growth, which in turn triggered the preferential degradation of fats and proteins as energy and nitrogen sources (Guo et al., 2025). Lipases catalyzed the hydrolysis of triglycerides into free fatty acids, directly leading to a sharp increase in acid value and a decrease in fat content. Proteases broke down storage proteins into peptides and amino acids, resulting in a decline in protein content.

SEM imaging revealed progressive microstructural degradation during fungal colonization. The control samples exhibited a tightly packed cellular architecture with a uniform distribution of proteins, starch, and lipids (Fig. 4e). During the initial infection stages (Fig. 4f), minor cytoplasmic disintegration was observed, indicating early hyphal penetration and cell wall disruption. By day 11 (Fig. 4g), severe cellular disruption was evident, with noticeable degradation of lipid bodies. In the advanced stages of infection (Fig. 4h, red circle), complete cellular collapse and nutrient depletion were observed, which correlated with significant deterioration in quality. *A. flavus* consumes nutrients such as lipids and proteins during its colonization of peanuts, leading to quality deterioration of the peanuts (Nie et al., 2024), which results in a loss of both nutritional and economic value. The complete cellular collapse and nutrient release observed in the advanced stages (Fig. 4h) likely provided abundant accessible nutrients (e.g., free fatty acids, amino acids) for *A. flavus*, which are not only essential for its growth but also serve as precursors or inducers for aflatoxin biosynthesis. The temporal correlation between biochemical changes and morphological damage provides valuable mechanistic insights into the processes leading to aflatoxin content.

### 3.3 Hyperspectral characteristics and spectral analysis

**Fig. 5:**
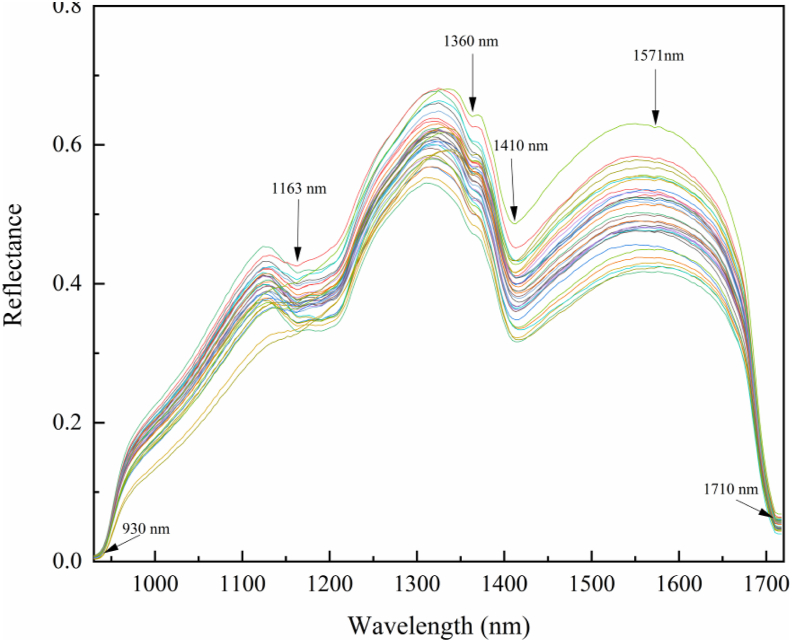
Raw hyperspectral profiles of peanut samples.

Hyperspectral images (HSI) were acquired across 224 spectral bands, ranging from 900 to 1700 nm. As shown in Fig. 5, the overall trends of the HSI curves were highly similar, but the peak intensities varied significantly depending on the degree of fungal contamination. The raw spectral data revealed that samples with low contamination levels exhibited the lowest average reflectance, while severely contaminated samples showed higher reflectance. The increase in reflectance in severely contaminated samples may be related to the proliferation of mold mycelium on the peanut surface. The scattering properties of the mycelium, as well as its inherent spectral characteristics, may alter the overall optical response of the samples. Additionally, changes in tissue structure and chemical composition resulting from lipid oxidation and protein denaturation are potential causes of the observed increase in reflectance. Key absorption peaks were observed at approximately 940 nm, 1163 nm, 1360 nm, 1410 nm, 1571 nm, and 1710 nm. The broad absorption regions near 940 nm and 1163 nm were primarily associated with C-H functional groups, specifically the second overtone of -CH_2_ and -CH_3_ stretching vibrations in lipids (Malik et al., 2024; Xi et al., 2025). The less pronounced peak at 1360 nm corresponded to the combination band of C-H bonds in cellulose (Hu et al., 2024), while the strong absorption feature near 1410 nm originated from the first overtone of O-H stretching vibrations (Luo et al., 2023). The absorption peak at 1570 nm was linked to amide II bands (Zhang et al., 2022), and the 1710 nm peak likely resulted from the first overtone of C-H stretching in proteins (Malik et al., 2024). Significant differences in nutrient content were observed between healthy and contaminated peanut kernels. The subtle spectral variations in these absorption peaks indicated AFB_1_ accumulation, further confirming the nutritional loss in *A. flavus*-infected peanuts. These findings suggest that HSI data, combined with toxin quantification, can be used to develop robust predictive models. However, since each HSI curve contains 224 wavelengths, the dataset may introduce computational complexity and noise interference in predictive modeling. Therefore, future research will focus on wavelength selection techniques to reduce data dimensionality while preserving critical spectral features for accurate contamination assessment.

### 3.4 Comparative analysis of spectral preprocessing methods

**Fig. 6:**
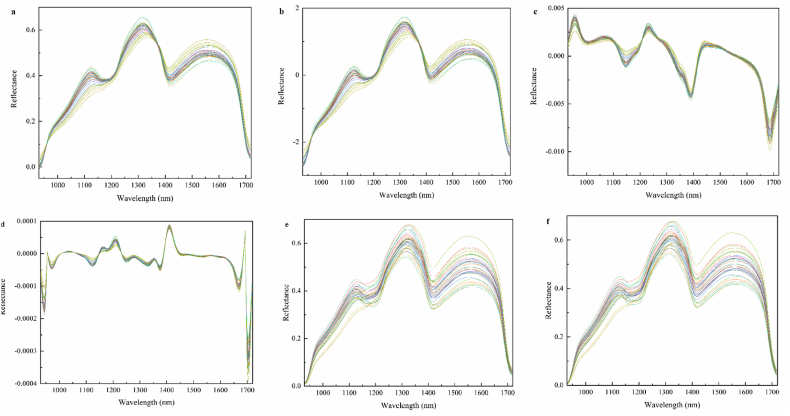
Spectral profiles after different preprocessing methods: (a) SNV (Standard Normal Variate), (b) MSC (Multiplicative Scatter Correction), (c) SG+1d (SG smoothing with 1st derivative), (d) SG+2d (SG smoothing with 2nd derivative), (e) WTD (wavelet transform denoising), (f) MF (median filtering).

Fig. 6 and Table 1 present a comprehensive comparison of six spectral preprocessing methods in terms of signal-to-noise ratio (SNR), spectral angle mapper (SAM), root mean square error (RMSE), coefficient of determination (R^2^), and fit time. The results demonstrate significant variations in the optimization efficacy of the different preprocessing approaches. MF exhibited superior noise reduction performance, achieving an exceptional SNR of 33.61 dB, which was substantially higher than that of the other methods. Notably, WTD also showed commendable denoising capability (SNR = 33.02 dB), though with significantly longer computational time (2.82 s) compared to MF (0.23 s). Regarding spectral fidelity, MSC yielded optimal SAM results (0.64°), consistent with its design principle of eliminating scattering effects. Most importantly, MF-preprocessed data demonstrated outstanding predictive performance, achieving the lowest RMSE (0.009) and the highest R^2^ (0.99). This suggests that MF is highly effective at preserving spectral features associated with aflatoxin content while efficiently suppressing noise. In summary, MF demonstrates excellent performance across all metrics, achieving the best noise reduction (SNR = 33.61 dB), prediction accuracy (R^2^ = 0.99), and spectral feature preservation (SAM = 1.2°), while also exhibiting optimal computational efficiency (0.23 s). This robust performance is likely attributed to MF's nonlinear characteristics, which allow for effective removal of impulse noise without excessive smoothing of critical spectral features (Zhou et al., 2022). Therefore, median filtering was selected as the standard preprocessing method for subsequent analyses, providing a reliable data foundation for the development of high-precision aflatoxin prediction models.

**Table 1:** Evaluation results of different spectral preprocessing methods.

Processing Method	SNR/db	SAM/°	RMSE	R^2^	Fit time/s
SNV	30.54	1.21	0.094	0.80	0.53
MSC	23.70	0.64	0.053	0.92	0.63
SG+1d	30.37	2.57	0.055	0.88	1.05
SG+2d	30.34	2.74	0.078	0.82	0.50
WTD	33.02	1.23	0.012	0.98	2.82
**MF**	**33.61**	**1.2**	**0.009**	**0.99**	**0.23**

SNV Standard Normal Variate, MSC Multiplicative Scatter Correction, SG+1d SG smoothing with 1st derivative, SG+2d SG smoothing with 2nd derivative, WTD Wavelet Transform Denoising, MF Median Filtering, SNR Signal-to-noise Ratio, SAM Spectral Angle Mapper, RMSE Root Mean Square Error, R^2^ Coefficient of Determination.

Bold indicates the optimal preprocessing methods.

### 3.5 Model evaluation

This study systematically compared the predictive performance of four modeling approaches—PLSR, RF, LASSO, and CNN—using both full-spectrum data and feature subsets selected by the SPA, SFLA, and GA algorithms (Table 2). Significant differences in predictive capability were observed among the models, with the CNN model, combined with GA feature selection, demonstrating superior performance. When using full-spectrum data, PLSR (R^2^v = 0.6181) and CNN (R^2^v = 0.6375) outperformed RF (R^2^v = 0.4026) and LASSO (R^2^v = 0.6023). The robustness of PLSR likely stems from its effectiveness in modeling linear relationships (Dong et al., 2023), while the competitive performance of CNN with full-spectrum data indicates its inherent ability to extract nonlinear features. In contrast, the suboptimal performance of RF and LASSO may be attributed to RF's susceptibility to overfitting with high-dimensional data and LASSO's instability when handling strongly correlated variables. Notably, GA-based feature selection consistently enhanced model performance across all approaches. The CNN model with GA feature selection achieved exceptional results (R^2^c = 0.9791, RMSEc = 7.1245, RPDc = 2.7864; R^2^v = 0.9845, RMSEv = 6.2032, RPDv = 2.8378), demonstrating the effectiveness of GA in identifying the most discriminative spectral features while mitigating overfitting. The study confirms that the CNN model with GA feature selection offers significant advantages in aflatoxin prediction in peanuts, with a high R^2^ (>0.98) and low RMSE (<7), indicating that this method can serve as an efficient and reliable nondestructive detection technology.

**Table 2:** Performance evaluation of different modeling methods using full-spectrum and selected featured bands.

Modeling methods	Feature selection methods	Training Set			Validation Set		
		R^2^	RMSE	RPD	R^2^	RMSE	RPD
PLSR	FULL	0.7453	21.5833	1.9870	0.6181	30.9884	1.6400
	SPA	0.8045	19.2554	2.1587	0.8114	18.7124	2.3336
	SFLA	0.700	25.0927	1.8324	0.6628	25.6223	1.7452
	GA	0.7476	21.3965	1.9960	0.6819	25.3013	1.7970
RF	FULL	0.5607	56.988	1.5307	0.4026	75.9781	1.3127
	SPA	0.6026	54.278	1.6127	0.6822	45.876	1.9618
	SFLA	0.6004	54.349	1.6103	0.6089	52.661	1.6905
	GA	0.7130	43.503	2.0532	0.7419	32.074	2.3179
LASSO	FULL	0.5556	44.7819	1.5220	0.6023	44.1257	1.5674
	SPA	0.5148	46.793	1.4565	0.5341	45.2513	1.4825
	SFLA	0.5197	46.5553	1.4640	0.5386	45.1034	1.4950
	GA	0.5362	45.7470	1.4899	0.5756	44.4145	1.5402
CNN	FULL	0.6784	19.7637	1.8885	0.6375	20.9072	1.2886
	SPA	0.7907	16.9221	2.0167	0.6425	20.0422	1.7629
	SFLA	0.8073	16.2766	2.1133	0.7546	17.1275	1.9393
	**GA**	**0.9791**	**7.1245**	**2.7864**	**0.9845**	**6.2032**	**2.8378**

PLSR Partial Least Squares Regression, RF Random Forest, LASSO Least Absolute Shrinkage and Selection Operator, CNN Convolutional Neural Network, FULL Full-spectrum, SPA Successive Projection Algorithm, SFLA Shuffled Frog Leaping Algorithm, GA Genetic Algorithm, R^2^ Coefficient of Determination, RMSE Root Mean Square Error, RPD Residual Prediction Deviation.

Bold indicates the optimal prediction model.

### 3.6 Correlation analysis

**Fig. 7:**
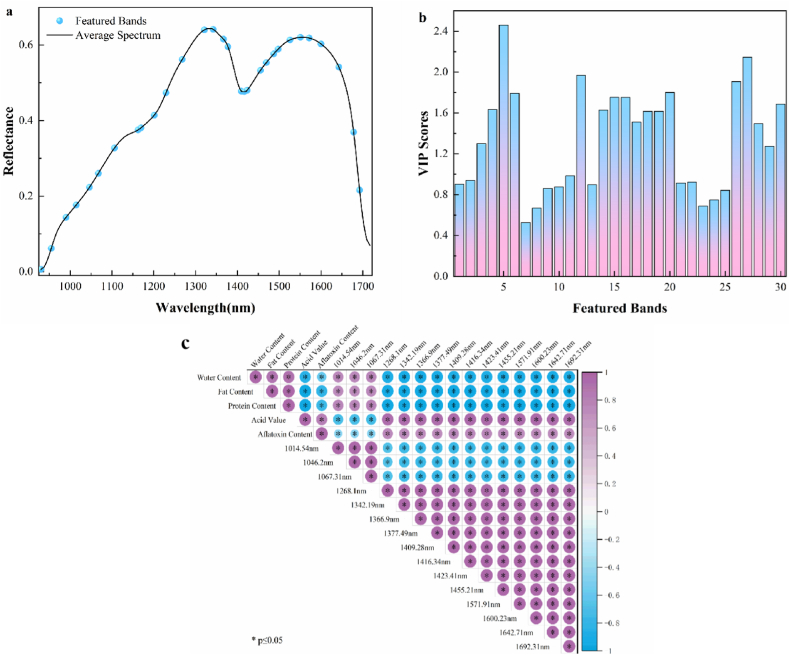
(a) Spectral curves with identified featured bands, (b) VIP (Variable Importance in Projection) scores of selected wavelengths, (c) Heatmap showing Pearson correlation coefficients among physicochemical properties, aflatoxin content, and featured bands.

GA selected thirty characteristic wavelengths, whose positions within the full spectral range are shown in Fig. 7a. The variable importance in projection (VIP) scores for these selected wavelengths are displayed in Fig. 7b, with the highest score observed at 1046.2 nm (VIP score = 2.4607), followed by 1600.23 nm and 1067.31 nm (VIP scores = 2.1458 and 1.7920, respectively). The absorption peak around 1046 nm may correspond to the third overtone of RNH_2_ stretching vibrations, which is typically associated with protein content. Additionally, unsaturated δ-lactone rings show absorption in the 1060–1270 nm range. Pearson correlation analysis was conducted to assess the relationships between the physicochemical properties of peanut kernels, aflatoxin content, and the top fifteen VIP-scored wavelengths selected by GA. As shown in Fig. 7c, significant positive correlations (r > 0.9, p ≤ 0.05) were found between moisture content, fat content, protein content, and aflatoxin concentration, while the acid value exhibited a significant negative correlation with toxin levels. Most of the featured bands showed strong positive correlations (r > 0.9) with all physicochemical properties, except for the acid value, as well as with aflatoxin content. These results further confirm substantial nutritional differences between healthy and *Aspergillus flavus*-contaminated peanuts, which manifest as variations in spectral absorption peak intensities. The established correlations between physicochemical parameters, characteristic wavelengths, and aflatoxin content suggest that these features could serve as reliable indicators for assessing peanut contamination levels. This finding provides a theoretical foundation for the development of rapid detection methods based on characteristic wavelength analysis.

### 3.7 Model prediction results

**Fig. 8:**
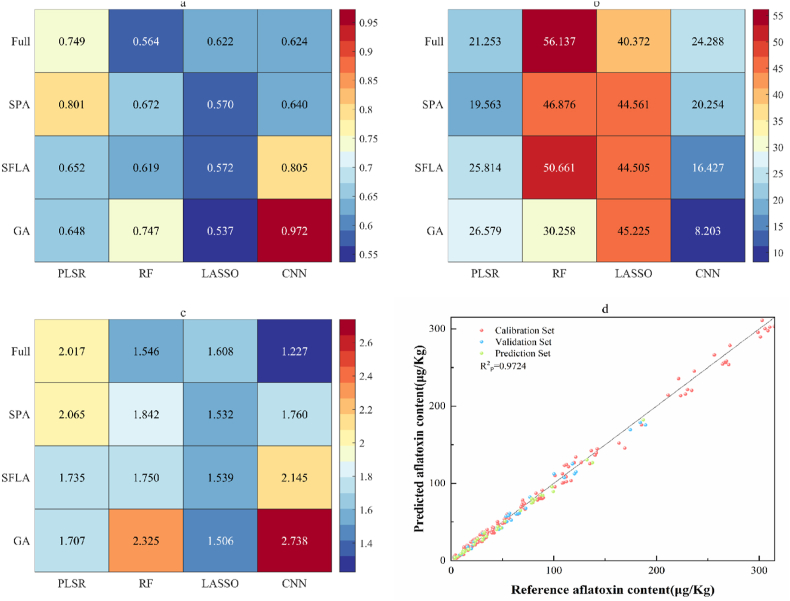
Predictive performance of different feature selection and modeling methods: (a) R^2^ values, (b) RMSE values, (c) RPD values; (d) Scatter plot of predicted versus measured aflatoxin content using GA-CNN model.

The machine learning algorithms mentioned above were applied to model the prediction set of peanut samples, with the results shown in Fig. 8a–c. To ensure a fair comparison, all machine learning and deep learning models were subjected to a comprehensive hyperparameter optimization process prior to evaluation on the test set. For traditional machine learning models, a grid search coupled with 5-fold cross-validation was employed to identify the optimal parameter set. For the CNN model, a random search strategy was utilized on a held-out validation set. Among the models tested, PLSR and CNN demonstrated superior predictive performance compared to RF and LASSO. Specifically, PLSR achieved better results when combined with SPA-based feature selection, while LASSO performed optimally with full-spectrum data, likely due to its inherent suitability for high-dimensional datasets. Notably, the CNN model with GA-based feature selection exhibited the best overall performance, achieving an R^2^p of 0.972, RMSEp of 8.203, and RPDp of 2.738. The regression scatter plot for this model (Fig. 8d) shows that all data points are closely distributed around the central line, indicating a strong linear relationship between the predicted and reference aflatoxin content. The CNN model's exceptional performance can be attributed to its powerful automatic feature extraction capability, which allows efficient capture of multi-level spatial-spectral features from hyperspectral data, facilitating the precise modeling of complex nonlinear relationships (Li et al., 2025). Unlike traditional algorithms (e.g., PLSR and RF), CNN employs local receptive fields and weight-sharing mechanisms through convolutional kernels to adaptively learn the most discriminative feature combinations. Furthermore, CNN's strong compatibility with high-dimensional data ensures robust performance, even with unfiltered full-spectrum data. In contrast, LASSO, despite its suitability for high-dimensional datasets, lacks the capacity for deep feature abstraction. The integration of GA optimization further enhanced CNN's feature selection efficiency by effectively eliminating redundant information, thereby improving model generalizability. These results confirm that HSI combined with CNN is a feasible and effective approach for aflatoxin detection in peanuts, offering high accuracy, reliability, and applicability for industrial quality control.

## 4 Conclusion

This study established a deep learning prediction model based on hyperspectral imaging (900–1700 nm) for detecting aflatoxin content in peanuts. Through comprehensive analysis of physicochemical properties and SEM observation of microstructural changes, we demonstrated that *A. flavus* contamination leads to significant alterations in the nutritional composition and cellular structure of peanuts, which in turn modify spectral absorption characteristics. These findings validate the feasibility of developing prediction models based on hyperspectral imaging (HSI) data and aflatoxin content. Our systematic investigation compared various preprocessing methods, feature selection algorithms, and modeling approaches, including conventional regression models and deep learning architectures. Correlation analysis further confirmed significant relationships between physicochemical parameters, toxin concentrations, and characteristic spectral bands. The modeling results showed that optimal predictive performance (R^2^p = 0.972, RMSEp = 8.203, RPDp = 2.738) was achieved using median filtering (MF) preprocessing, genetic algorithm (GA) feature selection, and convolutional neural network (CNN) modeling. This combination demonstrates that HSI coupled with CNN is an effective, rapid, and nondestructive method for aflatoxin detection in peanuts, with substantial potential for high-throughput field applications in production monitoring. However, the relatively small sample size in this study may affect the model's generalizability, and other challenges such as the limitation to a single variety and high equipment costs may restrict the future development of this research (Yang et al., 2025). Therefore, future research will aim to overcome these limitations, improve the model's robustness and generalizability, and fully utilize its advantages for practical application in the food industry's production processes. To translate this potential into practical application and further advance the research, several concrete directions are proposed for future work: (1) Expanding the scope of validation to include a wider range of peanut varieties and growth conditions is crucial to verify the model's robustness and generalizability. (2) Future efforts will focus on developing lightweight CNN architectures suitable for deployment on portable HSI systems for real-time, in-field detection. (3) Exploring the fusion of hyperspectral data with other sensing modalities could create a more powerful multi-modal predictive framework. (4) Ultimately, translating the most informative wavelengths identified by the model into a low-cost, filter-based multispectral imaging system represents the most promising path toward widespread industrial adoption.

## Ethical approval

Ethics approval was not required for this research.

## Author statement

We the undersigned declare that this manuscript is original, has not been published before and is not currently being considered for publication elsewhere.

We confirm that the manuscript has been read and approved by all named authors and that there are no other persons who satisfied the criteria for authorship but are not listed. We further confirm that the order of authors listed in the manuscript has been approved by all of us.

We understand that the Corresponding Author is the sole contact for the Editorial process. She is responsible for communicating with the other authors about progress, submissions of revisions and final approval of proofs.

## Declaration of competing interest

The authors declare that they have no known competing financial interests or personal relationships that could have appeared to influence the work reported in this paper.

## Data Availability

The data that support the findings of this study are available upon request by contact with the corresponding author.
