# Supplementary material for: High-throughput quantification of aflatoxin in moldy peanuts using hyperspectral imaging and CNN: Comparative evaluation of machine learning algorithms and deep learning models
Source: Curr Res Food Sci. 2025 Sep 24;11:101209. doi: 10.1016/j.crfs.2025.101209 (PMC12510075; doi:10.1016/j.crfs.2025.101209)
Supplement: Multimedia component 1 [file mmc1.docx]

**Table S1** **Parameters of PLSR model**

| **Model** | **Latent Variables** | **Method** | **K-fold** |
| --- | --- | --- | --- |
| PLSR | 15 | center | 10 |

*** PLSR Partial Least Squares Regression**

**Table S2** **Parameters of RF model**

| **Model** | **Number of Trees** | **Min Leaf Size** | **Features per Split** | **Max Evaluations** |
| --- | --- | --- | --- | --- |
| RF | 200 | 10 | 10 | 30 |

*** RF** Random Forest

**Table S3** **Parameters of LASSO model**

| **Model** | **Alpha (α)** | ****Cross-Validation**:** | ****Lambda Grid**** | ****MaxIter**** | ****Tolerance**** |
| --- | --- | --- | --- | --- | --- |
| LASSO | 0.7 | 3 | 50 | 1e5 | 1e-7 |

*** LASSO Least Absolute Shrinkage and Selection Operator**

**Table S4** **Parameters of CNN model**

| ****Model**** | ****Epoch Number**** | ****Mini-Batch Size**** | ****Initial Learning Rate**** | ****Dropout**** | ****Optimizer**** | ****Kernel Size**** | ****Filters**** |
| --- | --- | --- | --- | --- | --- | --- | --- |
| CNN | 200 | 16 | 0.001 | 0.3 | Adam | 1×3 | 16/32 |

*** CNN Convolutional Neural Network**
